# Hybrid Approach to Protein–Protein Complex Affinity Prediction Based on Language Models and Molecular Dynamics

**DOI:** 10.3390/ijms27135925

**Published:** 2026-06-30

**Authors:** Elizaveta A. Bogdanova, Artem V. Chernukhin, Alexey K. Shaytan

**Affiliations:** 1 AI Centre and Department of Biology, Lomonosov Moscow State University, Moscow 119991, Russia; bogdanovaea@my.msu.ru; 2 Department of Information and Computer Technologies, Mendeleev University of Chemical Technology of Russia, Moscow 125047, Russia; chernukhin.a.v@muctr.ru

**Keywords:** binding affinity prediction, protein–protein interactions, deep learning, protein language models, molecular dynamics, Voronoi tessellation, structural bioinformatics

## Abstract

Protein–protein and protein–peptide interactions are fundamental to biological processes, making the accurate prediction of their binding affinity crucial for drug design and mutational analysis. Here, we develop HyBind-NN, a multimodal graph neural network that integrates protein language models (PLMs) with 3D structural and dynamic datasets to predict protein–protein and protein–peptide affinity. First, we demonstrate that combining ESM-2 sequence embeddings with precise 3D Voronoi spatial geometry enables accurate affinity predictions across diverse structural datasets. Next, we show that the inherent limitations of static rigid-body structures can be mitigated through a multi-task learning framework. By utilizing residue-level root mean square fluctuations (RMSF) derived from molecular dynamics (MD) as an auxiliary training target, the model implicitly learns to capture the conformational entropy of flexible peptides without requiring computationally expensive MD simulations during inference. In our benchmarking study, we observe that this multimodal architecture outperforms both purely sequence-based and strictly structural state-of-the-art algorithms, achieving a mean absolute error of 0.89 for pK_D_ (1.12 kcal/mol for ∆G) on the independent benchmark. Finally, we confirmed through ablation analysis that while the PLM provides the dominant predictive signal, geometric representations and dynamic regularization are crucial for resolving subtle conformational rearrangements. This study highlights the synergistic potential of combining PLMs with physics-aware architectures and demonstrates their application towards the robust prediction of intermolecular binding affinity.

## 1. Introduction

Protein–protein (PPI) and protein–peptide interactions drive the vast majority of biological processes, from intracellular signaling and enzymatic catalysis to immune responses and disease pathogenesis. Quantifying thermodynamic binding affinity—typically expressed as the dissociation constant (K_D_) or the change in Gibbs free energy (∆G)—is crucial for biomedical applications [1,2]. This knowledge is essential for rational optimization of therapeutic monoclonal antibodies, the design of specific peptide inhibitors, and predicting the phenotypic consequences of somatic mutations [3,4,5].

Despite major advances in structural biology, measuring thermodynamic binding parameters in vitro (e.g., via isothermal titration calorimetry or surface plasmon resonance) remains labor-intensive and low-throughput [6]. Experimental screening of combinatorial libraries is physically limited. Consequently, specialized databases like SKEMPI 2.0 [7] capture only a fraction of possible mutational landscapes and are heavily biased toward destabilizing substitutions. Developing accurate, scalable, and physically interpretable computational methods to predict binding affinity and the effects of point mutations (∆∆G) is therefore a central challenge in structural bioinformatics [8].

Historically, computational approaches have bifurcated into sequence-based and structure-based models. The breakthrough in 3D structure prediction led by AlphaFold [9] has accelerated the application of deep learning in this domain. Protein language models (PLMs) like ESM-2 [10] and ProteinBERT [11], alongside zero-shot predictors [12], can now estimate protein functional properties directly from primary sequences [13]. Transfer learning platforms such as APPT [14], ProtT-Affinity [15], and BindPred [16] extract co-evolutionary and physicochemical patterns from contextual embeddings. Recent frameworks utilizing Parameter-Efficient Fine-Tuning project interacting sequences into a shared latent space, where cosine similarity correlates directly with experimental affinity [17]. Specialized NLP-inspired architectures like MINT [18], pre-trained on billions of contacts from the STRING database, further highlight the potential of sequence-based mutational effect prediction.

This limitation has spurred the development of geometric deep learning and 3D graph neural networks (GNNs) [19,20]. Recent architectures, including ProAffinity-GNN [21] and PCANN [22], model protein complexes as heterogeneous spatial graphs. Rather than relying on simple Euclidean distance thresholds, these methods utilize 3D Voronoi tessellation as a more accurate biophysical edge descriptor. Voronoi partitioning allows for the precise calculation of per-atom contact areas and local solvent-accessible surface area (SASA), effectively approximating hydrophobic effects and van der Waals interactions, as demonstrated by VoroIF-GNN [23] and DeepUMQA-PA [24]. Incorporating explicit geometric contact graphs has been shown to improve the predictive performance even of advanced end-to-end models like Boltz-2 [25].

To bridge the gap between sequence and structure, recent related architectures, such as eGRAL, successfully combine PLM embeddings with atomic- or residue-level graphs for binding affinity prediction [26]. Yet, despite their computational efficiency, the majority of these PLM-GNN and structure-centric models operate under static, rigid-body assumptions. HyBind-NN distinguishes itself from these related approaches by explicitly addressing the limitations of static topology. By fusing Voronoi spatial graphs with ESM-2 embeddings, and crucially incorporating conformational entropy into its latent space via dynamic multi-task regularization, HyBind-NN provides physics-aware affinity predictions for flexible complexes that static models typically fail to capture. Yet, static crystal structures represent only a single local minimum on a complex conformational energy landscape. Recent mega-scale studies on folding thermodynamics [27,28] emphasize that molecular flexibility is critical for target recognition. This is particularly relevant for peptides, where high conformational entropy dictates that interface dynamics must be explicitly considered. Furthermore, when evaluating mutational effects, structure-based computational methods frequently encounter the “rigid-body limitation”: in silico amino acid substitutions performed without local side-chain relaxation often generate artificial steric clashes or fail to capture novel cavity formations.

To bridge the persistent gap between static structural representations and thermodynamic reality, we introduce HyBind-NN, a novel physics-aware multimodal architecture. While previous state-of-the-art models typically treat sequence evolution, spatial geometry, and conformational dynamics as isolated predictive modalities, HyBind-NN distinctively fuses them into a single, cohesive framework. Rather than merely ensembling existing tools, our approach embeds the evolutionary depth of protein language models (ESM-2) directly into the strict topological constraints of 3D Voronoi graphs. The important methodological innovation of HyBind-NN lies in its multi-task learning framework: by utilizing molecular dynamics-derived root-mean-square fluctuations (RMSF) strictly as an auxiliary training target, the model explicitly encodes conformational entropy into its latent space. This architectural design allows the network to implicitly evaluate the flexibility of interacting molecules and predict binding affinity from a single static structure. Consequently, HyBind-NN eliminates the need for computationally expensive dynamic inputs during inference, offering a highly accurate and scalable solution that outperforms both purely sequence-based and strictly static structural algorithms.

## 2. Results and Discussion

### 2.1. Analysis of Data Heterogeneity and Error Distribution

**Figure 1:**
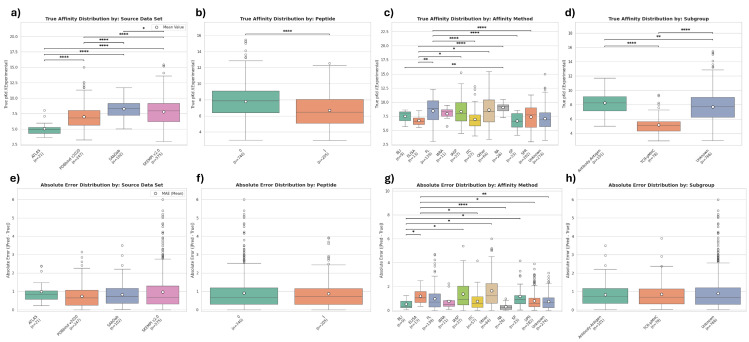
Distributions of experimental pK_D_ values (**a**–**d**) and absolute model prediction errors on the validation dataset (945 complexes) (**e**–**h**) stratified by various criteria: (**a**,**e**) source of the affinity data; (**b**,**f**) protein–protein (subgroup 0) versus protein–peptide (subgroup 1) complexes; (**c**,**g**) method of experimental affinity determination; and (**d**,**h**) known classes of the interacting molecules forming the complex. Statistical significance between independent groups was assessed using the two-sided Mann–Whitney U test. To account for multiple testing across subgroups, *p*-values were adjusted using the Benjamini–Hochberg False Discovery Rate procedure. Asterisks denote statistically significant differences: * *p*_adj_ < 0.05, ** *p*_adj_ < 0.01, **** *p*_adj_ < 0.0001.

The HyBind-NN model was trained for 50 epochs, and the checkpoint yielding the lowest mean squared error (MSE = 1.37) on the validation set was retained (Table S6). Because the validation set was used for model selection, it cannot provide an unbiased estimate of generalization performance. Nevertheless, given its substantial size and compositional diversity, we analyzed the distributions of both experimental affinities and prediction errors across various categories of complexes, stratified by data source, origin, and chemical nature (Figure 1).

The analysis of experimental affinity distributions (Figure 1) reveals substantial heterogeneity across various strata in the validation set. Stratification by data source highlights a distinct bias driven by the specific focus of each database (Figure 1a). For instance, ATLAS [29] provides affinity data for TCR-pMHC complexes, while SAbDab [30] focuses on antibody crystal structures. SKEMPI v2.0 primarily targets free energy changes upon point mutations, though the validation set still covers a broad affinity range for these complexes. The lowest mean absolute error (MAE ≈ 0.7) is observed for the SKEMPI v2.0 subset (Figure 1e). However, this subset contains significant outliers corresponding to structural hotspots that drastically alter binding geometry.

Substantial variance in true affinity is also evident across experimental measurement methods (Figure 1c). Techniques such as surface plasmon resonance (SPR), isothermal titration calorimetry (ITC), and biolayer interferometry (BLI) introduce inherent methodological noise (often reaching 0.5–1.0 kcal/mol) due to differing physicochemical conditions (e.g., buffer composition, pH, temperature) [31]. The error distribution stratified by affinity method (Figure 1g) highlights a fundamental limitation for computational approaches: absolute prediction errors vary significantly depending on whether SPR, BLI, ITC, or fluorescence spectroscopy was used. A predictive model cannot achieve accuracy exceeding the physical uncertainty of the experimental method used to generate its training targets. While incorporating experimental features like temperature and pH can partially mitigate this discrepancy, instrumental variance imposes a strict limit on prediction accuracy and remains a major component of the final error. The need for new, standardized experimental datasets has been similarly emphasized in other affinity prediction studies, such as PCANN.

**Figure A1:**
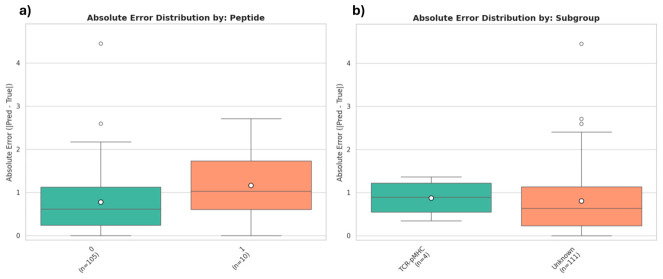
Distributions of absolute model pK_D_ prediction errors on Affinity Benchmark v5.5 dataset (n = 115) stratified by various criteria: (**a**) protein–protein (subgroup 0) versus protein–peptide (subgroup 1) complexes; (**b**) known classes of the interacting molecules forming the complex.

Data distribution also depends heavily on the conformational flexibility of the interacting molecules. A highly significant difference (*p* < 0.0001, effect size = 0.319) is observed between protein–protein (Peptide = 0) and protein–peptide (Peptide = 1) complexes (Figure 1b), with a noticeably lower median affinity for the latter. This aligns with standard biophysical principles: free peptides possess immense conformational entropy and frequently undergo folding-upon-binding transitions. Consequently, amino acid sequences and static structures alone (even after relaxation) are insufficient to fully capture these large-scale conformational rearrangements. Despite this challenge, HyBind-NN predicts peptide and protein affinities with comparable accuracy (MAE ≈ 0.7–0.8). This performance is achieved by integrating RMSF data derived from molecular dynamics trajectories, enabling the model to account for the conformational entropy of short chains during binding. This is further supported by the error distribution analysis for the Affinity Benchmark v5.5 [32] test set (Figure A1).

Among functional subgroups, affinity predictions for TCR-pMHC complexes exhibit the highest accuracy (MAE ≈ 0.6) and the tightest error distribution (Figure 1h), compared to antibody–antigen complexes and unclassified structures, where the mean error reaches ≈ 0.9. The language embeddings effectively encode both the highly conserved major histocompatibility complex scaffolds and the peptide variability within the binding groove. A similar trend is observed for TCR-pMHC complexes in the Affinity Benchmark v5.5 test set (Figure A1). Overall, analyzing absolute prediction errors across these strata reveals key biophysical patterns and underscores the fundamental limits of in silico modeling driven by experimental noise and molecular flexibility.

### 2.2. Testing and Ablation Analysis

**Figure 2:**
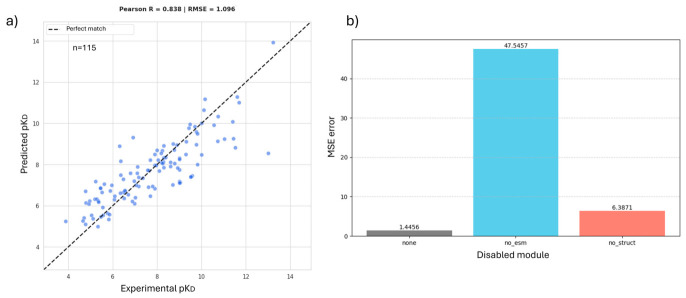
Performance evaluation of the predictive model on the independent Affinity Benchmark v5.5 dataset. (**a**) Scatter plot demonstrating the correlation between experimentally measured and predicted pK_D_ values. The dashed line indicates a perfect match, with the model achieving a Pearson correlation coefficient R of 0.84. (**b**) Ablation analysis illustrating the impact of individual architectural components on the overall mean squared error (MSE). The baseline model with all modules active (“none” disabled) is compared against variants lacking ESM language embeddings (“no_esm”) and structural features (“no_struct”).

To evaluate the generalization capability of the multimodal HyBind-NN algorithm, we used the independent Affinity Benchmark v5.5 test set (Table S7). Because these complexes were excluded from the training phase, they provide an unbiased assessment of de novo predictive performance. Figure 2a presents a scatter plot of the experimental versus predicted pK_D_ values. The model demonstrates strong agreement with the experimental data, achieving a Pearson correlation coefficient R of 0.84 and MAE of 0.89 for pKD (1.12 kcal/mol for ∆G). The largest prediction errors occur for complexes with pK_D_ > 10, which is largely driven by the underrepresentation of high-affinity complexes in the training dataset. This imbalance occurs because the training data relies heavily on the SKEMPI v2.0 database, which, as noted previously, primarily consists of destabilizing mutations derived from alanine scanning [33].

To quantify the contribution of each modality to predictive accuracy, an ablation study was conducted (Figure 2b). The full baseline architecture (“none”), which utilizes all available features (geometric and embeddings), achieves MSE of 1.4456. Removing the features extracted from the ESM-2 language model (“no_esm”) results in a severe degradation of performance, with the MSE increasing to 47.5457. This indicates that the evolutionary context and latent physicochemical properties of amino acid sequences act as the dominant predictors for estimating binding affinity.

Conversely, disabling the structural module (“no_struct”), which encompasses 3D Voronoi tessellation, yields a more than fourfold increase in error (MSE = 6.3871) relative to the baseline. Therefore, despite the primary importance of ESM-2, the integration of physical relaxation algorithms (e.g., FoldX [34]) and geometric graphs remains crucial for correctly resolving steric clashes and capturing the subtle conformational rearrangements induced by point mutations.

An ablation study was conducted during model training to isolate the contributions of PLM embeddings, the Voronoi network, FoldX energy parameters, and MD-derived dynamics (Table S1). To determine the specific impact of molecular dynamics, we evaluated a configuration that preserved the full 3D spatial architecture (Voronoi graph) but disabled the dynamics-related auxiliary objective (rmsf_head).

Omitting the MD auxiliary task increased the MSE by 1.51 and decreased the Pearson correlation coefficient from 0.81 to 0.74. This indicates that the model captures conformational entropy independently of the static geometric constraints provided by the Voronoi tessellation. Furthermore, removing FoldX-derived structural and energetic data reduced model performance, confirming that spatial geometry, energy parameters, and implicit molecular dynamics provide independent, orthogonal contributions to affinity prediction accuracy.

For a more objective evaluation of the developed model, we compared its performance against existing algorithms across several test sets. Table 1 presents a comparison between our architecture and state-of-the-art affinity predictors on the Affinity Benchmark v5.5 dataset. To ensure statistical robustness, 95% confidence intervals (CIs) were estimated using a bootstrapping procedure with 10,000 iterations. The evaluation on the Affinity Benchmark v5.5 dataset reveals that HyBind-NN consistently outperforms existing predictors in both accuracy and ranking capability. The proposed model achieved the lowest MAE of 0.81 pK_D_, with a narrow 95% CI of [0.68, 0.95]. The closest competing method, APPT [13], yielded a higher MAE of 0.97 (95% CI: [0.77, 1.12]), while the remaining baseline models (ProtT-Affinity [15], BindPred [16], and PPB-Affinity [33]) produced notably larger errors ranging from 1.28 to 1.36 pKD.

In addition to minimizing absolute prediction error, HyBind-NN demonstrated superior linear correlation with experimental values, reaching a Pearson correlation coefficient of 0.84. This substantially exceeds the correlation achieved by APPT (0.77) and represents a marked improvement over the other predictors (0.59–0.66). The tight confidence interval and high correlation coefficient confirm the stability and enhanced generalization of the HyBind-NN architecture, proving its efficacy in extracting predictive thermodynamic patterns on a diverse benchmark.

However, the margin of improvement over leading language models remains relatively modest. This suggests that currently available structural data is inherently noisy and insufficiently abundant to train predictive models more effectively—a conclusion further supported by the ablation analysis (Figure 2b).

**Figure 3:**
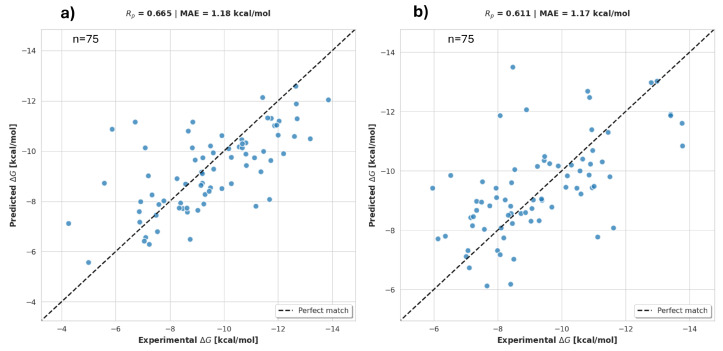
Correlations between predicted and measured Δ Gbind using datasets testA and testB. (**a**,**b**). The dashed line indicates a perfect match, with the model achieving a Pearson correlation coefficient R of 0.65 (**a**) and R of 0.56 (**b**).

To further benchmark the algorithm, it was evaluated on the Test A and Test B datasets (Table S8), which were originally developed to assess the PCANN [22] structural model. These test sets are absent from all training databases and contain affinity values obtained via ITC and SPR methods. Figure 3 illustrates the correlation between the experimental and predicted binding free energy changes (∆G, kcal/mol) for both datasets. The Pearson correlation coefficients were 0.66 for Test A (Figure 3a) and 0.61 for Test B (Figure 3b).

To elucidate the boundary conditions and systemic limitations of the HyBind-NN architecture, we isolated and structurally analyzed test-set complexes exhibiting absolute ∆G prediction errors greater than 3.0 kcal/mol. Detailed inspection revealed that these outliers predominantly represent non-standard interaction modes that violate the underlying physical assumptions of static, explicitly protein-only graph representations.

First, metalloproteins such as the human TFIIE complex (PDB ID: 5gpy) [35] and the Stn1-Ten1 complex (PDB ID: 4joi) [36] heavily rely on zinc-finger motifs and metal ion coordination to bridge the interaction interface. The necessary removal of heteroatoms during standard structural preprocessing forces the model to evaluate the interface based solely on the repulsive forces of uncoordinated polar or negatively charged residues, leading to severe underprediction of the binding affinity.

Second, several outliers heavily depend on post-translational modifications (PTMs). For instance, the yeast BRCT domain complex (PDB ID: 6j0y) typically recognizes phosphorylated targets, where the phosphate group provides a massive energetic contribution [37]. Similarly, the LRX8-RALF4 complex (PDB ID: 6tme) is significantly glycosylated, with glycans stabilizing the contact [38]. Because our current Voronoi tessellation strictly encodes the 20 canonical amino acids, the immense electrostatic and steric contributions of phosphates and carbohydrates are invisible to the network, resulting in an underestimated interface area.

Furthermore, the model struggled with integral membrane complexes, such as the SusC/SusD transporter (PDB ID: 5t3r) [39]. This limitation arises from the implicit water solvent assumptions inherent in standard energy terms (e.g., FoldX), which inevitably miscalculate hydrophobic interactions when applied to a lipid bilayer environment. Finally, complexes involving highly disordered viral peptides undergoing induced fit, such as the influenza endonuclease complexed with an NLS peptide (PDB ID: 5fml) [40], highlight the inherent challenge of estimating conformational entropy penalties from static graphs. Acknowledging these specific biological constraints confirms that the model operates correctly within its physical logic, while providing a clear roadmap for future iterations to incorporate explicit representations of non-protein cofactors, PTMs, and environmental contexts.

As detailed in Table 2, HyBind-NN achieved MAEs of 1.18 and 1.17 kcal/mol on Test A and Test B, respectively. The proposed algorithm statistically outperformed our previously developed ProBAN structural model [41] (1.63 and 1.66 kcal/mol), alongside other specialized architectures, including BindPPI [42] (1.40 and 1.43 kcal/mol) and PCANN (1.27 and 1.36 kcal/mol). CIs demonstrate the stability of the proposed approach. The error ranges for HyBind-NN ([0.96, 1.43] and [0.95, 1.41], respectively) are consistently shifted toward lower values compared to the closest baseline, PCANN (CI [1.03, 1.52] for Test A and [1.09, 1.57] for Test B).

Beyond reducing absolute error, HyBind-NN yields a more robust complex ranking. The Pearson correlation coefficients for our model reached 0.67 (Test A) and 0.61 (Test B), outperforming baseline models, including PCANN (0.58 and 0.45) and ProBAN (0.55 and 0.49). This indicates that the model is less susceptible to predicting toward the mean, a common limitation in regression tasks. The improved linear correlation reflects the stronger generalization capability of HyBind-NN, validating the architecture’s efficiency in extracting structural and thermodynamic patterns from independent datasets. This level of accuracy demonstrates the algorithm’s robustness when generalizing to novel protein interfaces, an improvement likely driven by the core innovation of HyBind-NN: the integration of molecular dynamics data into the training process.

In summary, the benchmarking results demonstrate the robust predictive performance and generalization capacity of the HyBind-NN architecture. By integrating ESM-2 sequence embeddings with Voronoi-based 3D geometry, the model yields lower prediction errors (MAE = 1.12 kcal/mol) on the Affinity Benchmark v5.5 than both strictly language-based and structure-based baselines. Furthermore, evaluation on the independent Test A and Test B datasets confirmed the algorithm’s stability when applied to novel protein interfaces. On these datasets, HyBind-NN outperformed well-known structural predictors, including PCANN and ProBAN, across all quality metrics analyzed. This increased accuracy is primarily driven by the core architectural addition of molecular dynamics features during training.

Despite these improvements, the relatively narrow performance gap between our multimodal approach and pure language models highlights a broader limitation in computational structural biology. The inherent noise and limited volume of currently available 3D structural datasets remain the primary bottlenecks, restricting the extent to which structure-guided in silico affinity predictors can be fully optimized.

### 2.3. Model Interpretability and Spatial Attention Analysis

To evaluate the model’s biological relevance and interpret its decision-making logic, we analyzed the spatial distribution of attention weights extracted from the hidden layers of the graph neural network. The GATv2 architecture dynamically evaluates the significance of intermolecular and intramolecular contacts (graph edges). Consequently, we calculated the aggregated “attracted attention” for each node (amino acid residue) by summing the weights of all incoming edges at the final graph layer. These values were normalized and mapped onto the temperature factor (B-factor) field in the PDB structures to generate a structural heatmap (S2, S3).

**Figure 4:**
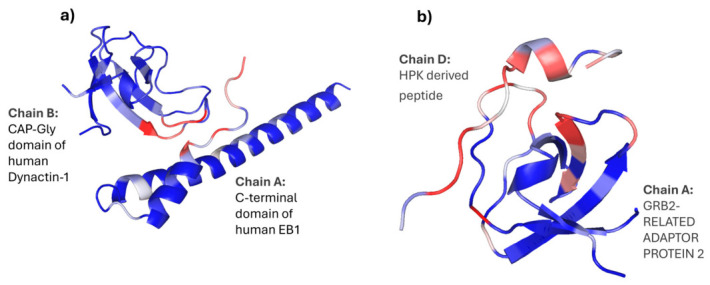
Visualization of graph attention weights for complexes from the test set. (**a**) Protein–protein complex 2HKQ (interaction between Dynactin-1 and EB1). (**b**) Protein–peptide complex 1UTI (GRB2 protein and a 16-amino acid HPK-derived peptide). Amino acid residues are colored according to their contribution to the final affinity prediction: from blue (low importance) through white to red (high importance).

Figure 4 illustrates the attention mapping for two representative complexes from the independent test set: the protein–protein complex 2HKQ (a) and the protein–peptide complex 1UTI (b). The color scale reflects the relative importance of residues for affinity prediction, ranging from blue (minimal attention) to red (maximum attention, or “hotspots”).

In complex 2HKQ (Figure 4a), representing the interaction between the CAP-Gly domain of dynactin-1 and the C-terminal domain of EB1, the highest attention weights are strictly localized at the structural interface. The network autonomously identifies the binding interface as the most critical region for predicting the free energy change, effectively disregarding distant, solvent-exposed residues (colored blue). A similar, though more pronounced, pattern is observed in the protein–peptide complex 1UTI (Figure 4b). Here, the adaptor protein GRB2 binds to a flexible 16-amino acid peptide derived from HPK kinase. The heatmap demonstrates that the network directs maximal attention toward both the peptide itself and the specific hydrophobic binding pocket on the target protein surface responsible for ligand anchoring.

This analysis confirms that the proposed architecture does not rely on dataset biases. By training on the geometric features of the Voronoi graph, the network successfully captures physically and biologically meaningful structural patterns, autonomously identifying critical topological regions and interaction hotspots without requiring explicit spatial annotations of the interacting functional groups.

## 3. Materials and Methods

### 3.1. Data Collection and Preparation

To construct the training and test sets, a comprehensive dataset of protein–protein and protein–peptide interaction structures was assembled. The PPB-Affinity dataset [33] served as the foundational collection. The initial dataset contained information on 12 062 interactions; using PDB identifiers and chain nomenclature, sequences were successfully extracted for 12 019 interactions. The final dataset aggregates data from the SKEMPI v2.0, PDBbind v2020 [43], SAbDab [30], Affinity Benchmark v5.5 [32], and ATLAS [29] databases, encompassing both wild-type complexes and amino acid variants featuring point mutations (Table S2). The combined dataset was deduplicated by comparing the sorted string hashes of the complexes’ amino acid sequences. In cases of duplication, priority was given to records with integrated MD data. Structures in which at least one chain was shorter than 50 amino acid residues were assigned a binary flag to investigate the specific characteristics of binding affinity prediction for protein–protein and protein–peptide complexes.

To integrate conformational flexibility data, we utilized 405 MD trajectories extracted from the DynaRepo database [44] and 127 trajectories from our previous work [41]. Following the final cleaning of the training data, 383 complexes with known trajectories remained for training and validation. A list of complexes with MD trajectories and a detailed protocol for calculating MD trajectories are described in the S1. Given potential inconsistencies in chain nomenclature between PDB structures and MD topologies, as well as the common truncation of flexible terminal regions during simulation preparation, a correspondence control algorithm was implemented. Initially, amino acid sequences were reconstructed from the structural topology JSON files and converted into a single-letter representation. Subsequently, the MD-derived sequences were mapped to the reference PDB structures using a pairwise alignment approach based on the Needleman–Wunsch algorithm. A structural match was confirmed only if the sequence identity exceeded 90% or if the sequence demonstrated full substring inclusion. Finally, for homo-oligomeric structures, redundant chains were discarded in favor of those achieving the highest alignment scores.

Three test datasets were utilized to evaluate the model. The first consisted of complexes extracted from Affinity Benchmark v5.5 that were not present in the training or validation sets. The second and third datasets (TestA and TestB) were sourced from the materials accompanying the previously developed PCANN algorithm. To prevent data leakage when using the ESM-2 language model, the division into training and test sets was performed using strict homology thresholds. Sequence identity was calculated using the MMseqs2 [45] software package. A complex was classified as highly identical to another complex only if all of its components exhibited a sequence identity of 30% or greater. Applying this criterion, the main test set, Affinity Benchmark v5.5, completely lacks complexes with an identity exceeding 30% relative to the training set. In the additional sets (Test A and Test B), only 7 complexes were identified as having an identity within the 40–50% range (Figure S1).

### 3.2. Structure Quality Control and Data Exclusion Criteria

Because the HyBind-NN algorithm relies on the precise three-dimensional geometry of intermolecular contacts derived from Voronoi tessellation, coordinate defects in PDB files can severely destabilize the training of graph convolutional layers. Experimental structures solved via X-ray crystallography or cryo-electron microscopy often contain disordered regions lacking electron density for specific atoms or entire amino acid residues. To address this, we implemented a multi-stage quality control and filtering protocol for the input structures:

1. Exclusion of complexes with interface backbone defects. Complexes lacking coordinates for heavy backbone atoms (N, Cα, C, O) within the binding interface (defined as inter-chain contacts within a 10Å radius) were removed. Missing Cα or Cβ atoms prevent the correct positioning of graph nodes and disrupt local Voronoi calculations, creating false discontinuities in the adjacency matrix and corrupting geometric edge descriptors;
2. Filtering by side-chain completeness. We assessed the presence of functional side-chain atoms for all residues at the intermolecular interface. Complexes were excluded if any key binding residue (“hot spot”) had missing atoms that could not be unambiguously reconstructed using standard rotamer libraries. This step is necessary because FoldX produces biophysically inaccurate relaxation energies when performing in silico mutagenesis on defective rigid-body structures;
3. Screening for unresolvable steric clashes. Both wild-type structures and their in silico-generated mutants were evaluated for van der Waals overlaps. If the clash score exceeded a threshold of 15 clashes per 1000 atoms after local energy minimization and side-chain relaxation in FoldX, the complex was discarded. This prevents severe computational artifacts from introducing numerical noise into the K_D_ predictions;
4. Sequence–structure mismatch filtering. We excluded complexes where the ESM-2 language model generated a continuous embedding vector for the full sequence, but the corresponding PDB file contained unresolved loops exceeding 5 consecutive residues at the interface. This ensured exact synchronization between the evolutionary and spatial data streams within the multimodal data loader.

**Figure 5:**
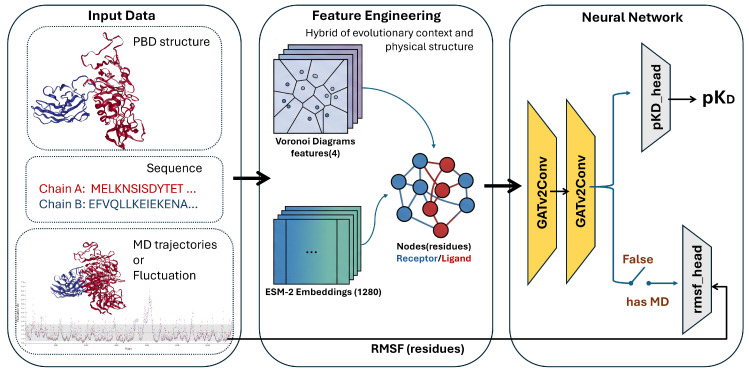
Complete computational pipeline for processing protein complexes and predicting binding affinity (pK_D_). The architecture utilizes multimodal Input Data, including static 3D coordinates (PDB structures), amino acid sequences, and dynamic residue fluctuations (RMSF) derived from molecular dynamics (MD) trajectories. During Feature Engineering, the algorithm constructs a residue-level graph of the receptor–ligand complex. Node representations are formed by concatenating evolutionary context—extracted via ESM-2 language embeddings (1280 dimensions)—with spatial and geometric properties derived from Voronoi tessellation (4 features). In the Neural Network module, the integrated graph is processed by two sequential Graph Attention Network (GATv2Conv) layers. The architecture employs dual output heads: the primary pKD_head predicts the binding affinity, while an auxiliary rmsf_head models residue flexibility. A conditional switch (“has MD”) allows the network to bypass the auxiliary head if trajectory paths are missing, ensuring that samples lacking MD data can still be processed and evaluated for affinity without being discarded from the dataset.

While this stringent filtering reduced the overall dataset size, it guaranteed the construction of physically valid and topologically continuous interaction graphs for every sample, substantially improving the generalization capability of the final neural network (Figure 5).

### 3.3. Graph Construction and Voronoi Geometry Calculation

To analyze structural characteristics, the complexes were formalized as undirected spatial graphs Ϟ = (V, E). The node set υ_i_ in V corresponds to amino acid residues, with their spatial positions defined by the coordinates of their Cα-atoms (*x_i_* in R^3^). For mutant complexes, a prior physical relaxation of side chains was performed using the FoldX algorithm.

Protein packing topology was modeled using a 3D Voronoi tessellation, where space is partitioned into non-overlapping cells *Ri*, defined by the condition 1:

(1) $$R_{i}=\{x\in R^{3}|{\left\|x-x_{i}\right\|}_{2}\leq {\left\|x-x_{j}\right\|}_{2},\forall_{j}\neq i\}.$$

An edge is established between two nodes if their respective Voronoi cells share a common face, subject to distance threshold constraints d_ij_ of less than 8.0 Å for intra-chain interactions and less than 10.0 Å for inter-chain interactions.

Each edge e_ij_ ∈E is represented by a 4D tensor 2:

(2) $$e_{ij}=\left[d_{ij},\frac{1}{d_{ij}+\epsilon },\;A_{ij},\delta_{ij}\right],$$

where d_ij_ denotes the Euclidean distance, and the term ($d_{ij}+\epsilon$) (with a smoothing constant ϵ = 10^−6^) approximates the decay of physical interactions. The parameter A_ij_ corresponds to the surface area of the Voronoi face, calculated via the cross product of its vertices, while δ_ij_ acts as a binary indicator of the contact type, where δ_ij_ = 1 for inter-chain contacts and δ_ij_ = 0 for intra-chain contacts.

### 3.4. ESM-2 Embeddings

The feature representation for each amino acid residue (graph node) was constructed by integrating evolutionary, contextual, and structural data. To obtain high-level characteristics of the complex’s amino acid sequences, we utilized the pre-trained ESM-2 protein language model (version: esm2_t33_650M_UR50D). The ProtT5 [46] model was also tested for generating embeddings; however, it demonstrated lower performance, similar to other ESM-2 variants (Table S3). Embeddings were extracted from the final hidden layer of the model for each residue. This approach captured evolutionary conservation, local secondary structure information, and the physicochemical properties of amino acids, providing a robust functional description of the residues even in cases where local 3D geometry provided insufficient information. To explicitly distinguish the roles of the interacting molecules, a binary chain-membership flag (one-hot encoding) was concatenated to the 1280-dimensional ESM-2 embedding vector. Utilizing a (1, 0) vector for the receptor and a (0, 1) vector for the ligand/peptide enabled the neural network architecture to adapt attention weights based on the specific functional role of each molecule within the complex. The full description of the data object structure is available in Table S4.

### 3.5. HyBind-Neural Network Architecture

The model is based on second-generation graph attention networks (GATv2). Unlike standard GAT architectures, the GATv2 layer employs dynamic attention coefficient computation, enabling the receiver node to generate a non-linear response to the properties of the ligand node. The attention coefficients α_ij_ are computed as follows:

(3) $$e(i,j)=v^{T}LeakyReLU(W\left[h_{i}\left\|h_{j}\right\|e_{ij}\right]),$$

(4) $$\alpha_{ij}={Softmax}_{j}(e(i,j)).$$

To optimize computational efficiency, the architecture was configured with a double attention head and a hidden dimension of 128. All hyperparameters and training details are presented in Table S4.

The network was trained using a multi-task learning framework comprising two objectives. The local task (RMSF Head) is implemented via a fully connected layer that predicts the RMSF for each Cα-atom. The global task (pK_D_ Head) utilizes an MLP block that processes the aggregated graph vector, obtained via global mean pooling, to predict the binding affinity constant pK_D_ (pK_D_ = −lg(K_D)_).

Given the heterogeneity of the dataset, where MD profiles are unavailable for a subset of complexes, a dynamic loss masking mechanism was implemented. The total objective function L_total_ is defined as 5:

(5) $$L_{total}=L_{affinity}(y,\hat{y})+\lambda \cdot \frac{1}{B}\sum_{b=1}^{B}(I({has\_md}_{b})\cdot \frac{1}{N_{b}}\sum_{i=1}^{N_{b}}{\left(r_{bi}-{\hat{r}}_{bi}\right)}^{2}),$$

where L_affinity_ represents MSE of the affinity prediction for a batch of size B; N_b_ denotes the number of nodes in graph b; $r_{bi}$ and ${\hat{r}}_{bi}$ represent the experimental and predicted RMSF values, respectively, and λ is a balancing hyperparameter. The indicator function I(has_md_b_) effectively gates the gradients from the RMSF head for complexes lacking MD data, allowing the shared network weights to be updated based solely on the global objective function when local structural information is unavailable (Figure 5).

## 4. Conclusions

In conclusion, the development of the HyBind-NN architecture successfully demonstrated the synergistic potential of integrating ESM-2 language embeddings with the precise spatial topology of 3D Voronoi graphs. A key achievement of this multimodal approach was the integration of molecular dynamics data—specifically residue-level RMSF—through a multi-task learning framework. By utilizing RMSF as an auxiliary training objective, the algorithm effectively learned to implicitly account for the conformational entropy of short chains without relying on dynamic inputs during inference. This proved especially advantageous for modeling highly flexible peptide interactions. Consequently, the model exhibited strong generalization capabilities on independent datasets, including Affinity Benchmark v5.5, Test A, and Test B, achieving a low MAE of 1.12 kcal/mol and outperforming established structural and language-based predictors such as PCANN, APPT, and ProBAN.

Despite these advances, several limitations remain. The performance margin between this hybrid model and pure language models was relatively modest, underscoring that the inherent noise and insufficient volume of currently available 3D structural data continue to bottleneck in silico geometric approaches. Furthermore, the model exhibited larger prediction errors for highly stable complexes (pK_D_ > 10), primarily because the foundational training databases, such as SKEMPI 2.0, are heavily biased toward destabilizing alanine-scanning mutations. Additionally, the fundamental physical uncertainty and methodological noise introduced by varied experimental measurement techniques (e.g., SPR, ITC, and BLI) impose a strict upper limit on the ultimate accuracy that computational models can currently achieve. The binding affinity metrics aggregated in large-scale databases, such as PDBbind and the Affinity Benchmark, were originally measured under widely varying in vitro conditions, including significant differences in temperature, pH, buffer composition, and ionic strength. Because intermolecular binding thermodynamics are highly sensitive to these environmental factors, treating these diverse values as standardized target variables inevitably introduces a degree of systematic noise into the training process. Currently, HyBind-NN evaluates affinity based strictly on geometric, energetic, and dynamic features, operating under an implicit assumption of uniform physiological conditions. Consequently, the algorithm cannot account for affinity shifts caused by local pH changes or specific salt concentrations. Future iterations of structure-based predictive models must aim to explicitly integrate these thermodynamic experimental variables as metadata inputs to accurately calibrate predictions according to specific solvent environments.

To address these challenges, future work will focus on augmenting the training datasets by incorporating artificial structures generated by advanced predictive models, such as AlphaFold 3 [47], to serve as robust positive and negative controls during training. We also plan to develop an independent, adapted functional module explicitly designed to analyze the specific thermodynamic contributions of single-point mutations (∆∆G). Finally, we intend to implement specialized predictive pipelines optimized for the distinct structural features and binding interfaces of antibody–antigen complexes, further expanding the applicability and precision of the HyBind-NN model.

## Figures and Tables

**Table 1:** Summary of the evaluation of HyBind-NN and other predictors on the dataset Affinity Benchmark v5.5.

Model	MAE (pK_D_)	95%CI	Pearson Corr
PPB-Affinity	1.36	[1.21, 1.59]	0.66
APPT	0.97	[0.77, 1.12]	0.77
BindPred	1.3	[1.17, 1.57]	0.6
ProtT-Affinity	1.28	[1.14, 1.61]	0.59
**HyBind-NN**	0.81	[0.68, 0.95]	0.84

**Table 2:** Summary of the evaluation of HyBind-NN and other predictors on Test A and Test B.

Model	MAE, kcal/mol	95%CI	Pearson Corr	MAE, kcal/mol	95%CI	Pearson Corr
	Test A			Test B		
ProBAN	1.63	[1.08, 1.85]	0.55	1.66	[1.13, 1.91]	0.49
BindPPI	1.40	[1.14, 1.63]	0.57	1.43	[1.16, 1.69]	0.43
PCANN	1.27	[1.03, 1.52]	0.58	1.36	[1.09, 1.57]	0.45
**HyBind-NN**	1.18	[0.96, 1.43]	0.67	1.17	[0.95, 1.41]	0.61

## Data Availability

The curated datasets used to train/validate and test the HyBind-NN model, including all specific dataset splits, are available on our GitHub at https://github.com/EABogdanova/HyBind-NN, accessed on 28 May 2026. The foundational raw datasets were acquired from the following publicly available databases: the raw data from PPI-Affinity is available at https://github.com/ChenPy00/PPB-Affinity, accessed on 28 May 2026; SKEMPI v2.0 is available at https://life.bsc.es/pid/skempi2, accessed on 28 May 2026; PDBbind v2020 is available at https://pdbbind-plus.org.cn/, accessed on 28 May 2026; SAbDab is available at https://opig.stats.ox.ac.uk/webapps/newsabdab/sabdab/, accessed on 28 May 2026; Affinity Benchmark v5.5 is available at https://zlab.umassmed.edu/benchmark/, accessed on 28 May 2026; and the ATLAS database is available at http://atlas.wenglab.org/, accessed on 28 May 2026. The raw molecular dynamics trajectories were sourced from DynaRepo, which is available at https://dynarepo.inria.fr. The source code used to train the HyBind-NN architecture to predict protein–protein and protein–peptide binding affinity, generate the 3D Voronoi graph representations, and integrate ESM-2 sequence embeddings, and a comprehensive guide to reproducing the benchmarking results demonstrated herein is available on our GitHub at https://github.com/EABogdanova/HyBind-NN, accessed on 28 May 2026.

## Supplementary Materials

The following supporting information can be downloaded at: https://www.mdpi.com/article/10.3390/ijms27135925/s1.
