# Supplementary material for: Hybrid Approach to Protein–Protein Complex Affinity Prediction Based on Language Models and Molecular Dynamics
Source: Int J Mol Sci. 2026 Jun 30;27(13):5925. doi: 10.3390/ijms27135925 (PMC13361561; doi:10.3390/ijms27135925)
Supplement: Supplementary file 1 [file ijms-27-05925-s001.zip › Supplementary S1.pdf]

Supplementary materials S1

Table S1. Ablation study of model components on the test set. The baseline model utilizes all structural, energetic, and dynamic features. Each subsequent row represents the isolated removal of a specific module.

Model Configuration	Pearson Corr	MSE
**Full Model**	**0.81**	**1.37**
w/o FoldX optimization	0.78	1.56
w/o MD auxiliary target	0.74	1.51
w/o Voronoi graph	0.69	1.59
w/o Voronoi and MD (*no_struct*)	0.63	1.77

Table S2. Composition of the training and test sets

Dataset Split	Data Source(s)	Number of Complexes
**Training Set**	PDBbind v.2020, SKEMPI 2.0, ATLAS, SAbDab	8926
**Validation Set**	PDBbind v.2020, SKEMPI 2.0, ATLAS, SAbDab	945
**Test 1**	Affinity Benchmark v5.5	115
**Test A**	PCANN	75
**Test B**	PCANN	75

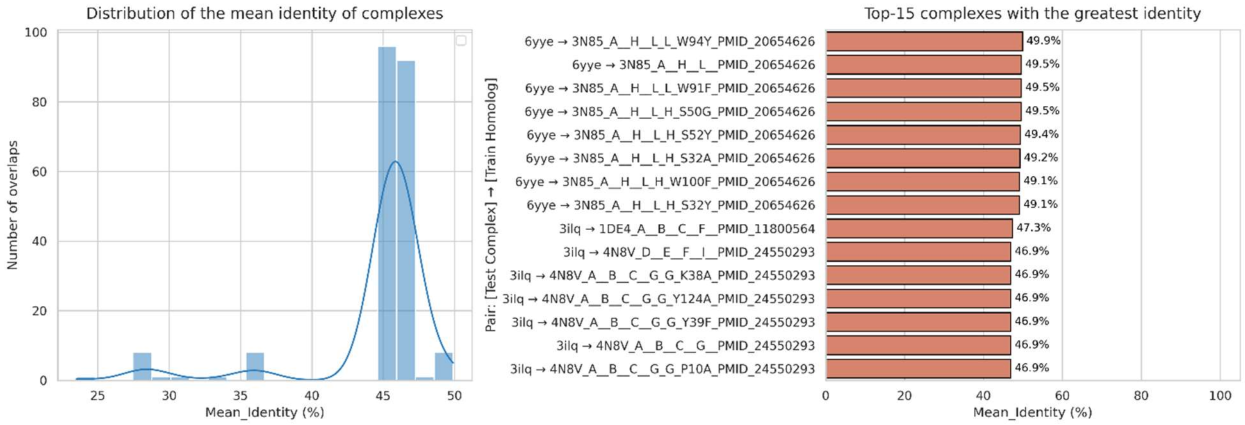

Figure S1. Evaluation of sequence homology between the independent test set and the training dataset. (Left) Distribution of the mean sequence identity for overlapping test-train complex pairs. The histogram demonstrates that the sequence identity for identified homologs strictly adheres to the predefined threshold, with no pairs exceeding 50%. (Right) A detailed bar chart highlighting the top 15 test-train pairs exhibiting the highest sequence identity. The maximum observed mean identity is 49.9% (e.g., between test complex 6yye and its training homolog 3N85),

confirming that a rigorous homology filter was successfully maintained to prevent data leakage and ensure an unbiased evaluation of the model's generalization capabilities

Table S3 Test results on the validation set for a model trained on different embeddings

**Embeddings type**	**MSE**	**Pearson corr**
esm2_t6_8M	2.81	0.41
esm2_t12_35M	1.76	0.43
esm2_t30_150M	1.54	0.54
esm2_t33_650M	1.37	0.81
esm2_t36_3B	1.39	0.83
ProtT5-XL-U50	1.43	0.78

Data object structure

The structure of the .pt object (which typically represents an instance of `torch_geometric.data.Data`) containing all information regarding the protein-protein complex within the HyBind-NN pipeline, is designed to store multimodal data: structural geometry, evolutionary features derived from ESM-2, and characteristics obtained from molecular dynamics.

Table S4 Data structure (PyTorch Geometric object)

**Attribute**	**Data Type**	**Description**
**x**	torch.Tensor	Node feature matrix. Dimensions: [N, 1282], where N is the number of amino acids. It includes a 1280-dimensional ESM-2 vector and 2 parameters indicating membership in a receptor or ligand.
**pos**	torch.Tensor	Spatial coordinates. Dimensions: [N, 3]. Coordinates of Cα atoms.
**edge_index**	torch.LongTensor	Edge (connection) indices. Size: [2, E]. Defines the graph topology (which node is connected to which).
**edge_attr**	torch.Tensor	Edge attributes. Size: [E, 4]. A 4-component tensor: $\left[d_{ij}, \frac{1}{d_{ij}+\epsilon}, A_{ij}, \delta_{ij}\right]$
**y**	torch.Tensor	Affinity Target. A scalar (or vector) of pK_D values for the entire complex.
**y_rmsf**	torch.Tensor	Node dynamics target. Size: [N, 1]. RMSF (fluctuation) value for each amino acid, extracted from MD trajectories.

Attribute	Data Type	Description
**has_md**	torch.BoolTensor	MD data availability flag. Scalar (True/False). Determines whether gradients for y_rmsf need to be masked.

Table S5 Training details and hyperparameters

Parameter	Value / Description
GPU	NVIDIA RTX A5000 (24 GB VRAM)
System RAM	64 GB
Node feature dimension	1282
Edge feature dimension	4
Global feature dimension	3
Hidden dimension	128
GNN Layers	2-times GATv2Conv
Dropout rate	0.2 (applied to fully connected layers)
Optimizer	Adam
Learning Rate	10^{-4}
Loss Function	Mean Squared Error (MSE)
Base Batch Size	4
Gradient Accumulation Steps	8 (Batch Size = 32)
Max Epochs	50

Protocol of MD simulation

Molecular dynamics simulations were performed using GROMACS 2024.2 with the CHARMM36m force field. The systems were solvated in a dodecahedral box using TIP3P water molecules (12 Å buffer) and 150 mM NaCl. Hydrogen atoms and histidine protonation states were assigned using the Reduce program.

Following energy minimization (steepest descent algorithm, 5000 steps), equilibration was conducted in the NPT ensemble (0.375 ns, 310 K, 1 bar; Berendsen thermostat and barostat) with a gradual, six-stage release of restraints on protein heavy atoms.

For each system, three independent 500 ns runs (2.0 fs timestep) were performed in the NPT ensemble at 310 K (V-rescale thermostat) and an isotropic pressure of 1 atm (C-rescale barostat). Bonds involving hydrogen atoms were constrained using the LINCS algorithm, and electrostatic interactions were calculated using the PME method. System coordinates were saved every 100 ps.

Fluctuation analysis calculates the root-mean-square fluctuation (RMSF) of atomic positions over the simulation time, following alignment to the initial frame.

List of complexes from the training and validation sets for which MD trajectory data were available:

1AK4, 1AVA, 1B2S, 1DP5, 1E96, 1EMV, 1F3V, 1FC2, 1FFW, 1FLT, 1FSK, 1GO4, 1GUA, 1I51, 1I5K, 1I8L, 1ICF, 1J7D, 1JTD, 1KAC, 1KGY, 1KXT, 1L2W, 1LFD, 1LJ2, 1LP1, 1LX5, 1LZW, 1MQ8, 1MTN, 1MXE, 1NCA, 1OP9, 1OQE, 1P27, 1PK1, 1PVH, 1PXV, 1RLB, 1RY7, 1S1Q, 1SV0, 1SYQ, 1T63, 1TA3, 1TDQ, 1TE1, 1U0S, 1UEX, 1UUZ, 1VET, 1W1W, 1WQJ, 1WR6, 1WRD, 1WVE, 1X75, 1XD3, 1XG2, 1XWD, 1YCS, 1YD8, 2AEQ, 2AW2, 2AZE, 2B7C, 2BDN, 2BO9, 2BP7, 2BYK, 2C0L, 2C7N, 2CJS, 2D10, 2G45, 2G9H, 2GH0, 2HRK, 2HTH, 2HWN, 2I32, 2I3T, 2IFF, 2IJ0, 2IY0, 2IYB, 2J0T, 2J8S, 2JIX, 2JJT, 2JKI, 2NQD, 2O3B, 2OIN, 2OJE, 2OMY, 2P4A, 2PCB, 2PJY, 2PMS, 2PSM, 2PTT, 2Q0O, 2QEJ, 2QHO, 2QXV, 2V1D, 2V3B, 2V5Q, 2V8S, 2V9T, 2VYR, 2W80, 2W8B, 2WD5, 2WO3, 2WP0, 2WUS, 2WX0, 2X89, 2XBB, 2XQR, 2XZE, 2Y5B, 2YBR, 2YGG, 2YVJ, 2Z8W, 2ZXX, 3B08, 3BH6, 3BIK, 3C66, 3C9A, 3CQX, 3DGC, 3DXC, 3E2U, 3EGG, 3EJH, 3EOY, 3F4Y, 3FF8, 3FHC, 3FXD, 3G3A, 3G9W, 3GJ8, 3GS2, 3GTY, 3H7B, 3H8D, 3HAE, 3HG0, 3HPW, 3HTU, 3HUG, 3IA3, 3IT8, 3K1R, 3K2M, 3K6G, 3K9M, 3KNB, 3LK2, 3NVN, 3NVQ, 3O4O, 3O5T, 3O6Q, 3OAK, 3OGO, 3ONA, 3ONL, 3ONW, 3OWT, 3P8M, 3P9W, 3QBT, 3QHT, 3QML, 3QQ8, 3QT2, 3QWQ, 3RZW, 3S5L, 3SYN, 3T0Y, 3TZ1, 3U82, 3UL4, 3UYO, 3UZ0, 3VEP, 3VUX, 3VV2, 3VYR, 3VYS, 3VZA, 3WA0, 3WN7, 3WWQ, 3ZEU, 3ZRZ, 3ZU7, 3ZWH, 3ZWL, 4AOQ, 4AQE, 4AYE, 4BKX, 4BQD, 4BRU, 4BWQ, 4C1N, 4C99, 4C9B, 4CC4, 4CJ2, 4D2G, 4DBG, 4DCN, 4DVG, 4EEF, 4EIZ, 4EOZ, 4EQA, 4ETP, 4ETW, 4EXP, 4FAO, 4FQ0, 4G8A, 4GEQ, 4GH7, 4GN4, 4H2W, 4H5S, 4HDO, 4HEP, 4HFK, 4I0C, 4IHH, 4ILW, 4J2L, 4JO6, 4JW3, 4K1R, 4K5A, 4KR0, 4KT5, 4LGP, 4LLO, 4LRX, 4LRZ, 4LYL, 4LZX, 4M0W, 4M5F, 4MP0, 4MQV, 4MRT, 4N7V, 4NG2, 4NIQ, 4NL9, 4NSO, 4OYD, 4PJ2, 4PW9, 4Q5E, 4Q96, 4QLP, 4REY, 4U2X, 4UDM, 4UU9, 4V0O, 4W4L, 4WNN, 4XKH, 4XL5, 4XVP, 4XWJ, 4Y61, 4YEB, 4YIQ, 4YN0, 4Z80, 4ZGY, 4ZQU, 4ZRJ, 4ZRK, 4ZW2, 5B78, 5BQE, 5CTR, 5CX3, 5CZF, 5DJU, 5DMJ, 5DOB, 5E3E, 5E6J, 5E7F, 5EB1, 5ELU, 5ET1, 5F1B, 5F5O, 5F5S, 5FZT, 5GJK, 5IMY, 5IP4, 5IUS, 5J4A, 5J57, 5J7C, 5JDO, 5JEM, 5JZE, 5KOF, 5KWY, 5KY5, 5L21, 5LXQ, 5M2M, 5MJY, 5ML9, 5N48, 5N88, 5NH3, 5NL1, 5O0W, 5OMN, 5OTJ, 5SXP, 5TGH, 5TL7, 5TVQ, 5UN7, 5UPK, 5UZU, 5W9A, 5WB7, 5WQL, 5WUJ, 5XQZ, 5YIR, 5YOY, 5Z7L, 6A6I, 6A7V, 6B12, 6B6U, 6BB4, 6BX5, 6C48, 6CO2, 6D13, 6DNQ, 6F3Z, 6FC3, 6FG8, 6FGO, 6FHP, 6FTO, 6FUB, 6GHO, 6H3T, 6H71, 6H9N, 6HUL, 6IM9, 6IVZ, 6J4S, 6J9L, 6JCS, 6JHW, 6M9K, 6ON9, 6OV2
